# Supplementary material for: Effects of acute stress on biological motion perception
Source: PLoS One. 2024 Sep 18;19(9):e0310502. doi: 10.1371/journal.pone.0310502 (PMC11410201; doi:10.1371/journal.pone.0310502)
Supplement: S1 Appendix — (DOCX) [file pone.0310502.s001.docx]

Table1 Stimulus set about multiplication problems in the practice experiment

| Serial number | Multiplication problems | Serial number | Multiplication problems |
| --- | --- | --- | --- |
| 1 | 2.97*3.19 | 6 | 1.95*5.37 |
| 2 | 2.98*3.18 | 7 | 1.96*5.34 |
| 3 | 2.99*3.17 | 8 | 1.97*5.28 |
| 4 | 2.99*3.51 | 9 | 2.37*3.98 |
| 5 | 2.98*3.52 | 10 | 1.95*4.87 |

Table2 Stimulus set about multiplication problems in the formal experiment

| Serial number | Multiplication problems | Serial number | Multiplication problems | Serial number | Multiplication problems | Serial number | Multiplication problems |
| --- | --- | --- | --- | --- | --- | --- | --- |
| 1 | 1.39*6.83 | 51 | 2.62*3.63 | 101 | 1.84*5.68 | 151 | 2.46*4.27 |
| 2 | 1.39*7.11 | 52 | 2.64*3.61 | 102 | 1.48*7.13 | 152 | 2.68*3.91 |
| 3 | 1.16*8.26 | 53 | 2.15*4.41 | 103 | 2.31*4.54 | 153 | 1.35*7.73 |
| 4 | 1.31*7.34 | 54 | 2.32*4.17 | 104 | 2.25*4.65 | 154 | 3.17*3.31 |
| 5 | 1.21*7.86 | 55 | 2.45*3.87 | 105 | 1.51*6.93 | 155 | 2.37*4.43 |
| 6 | 2.63*3.62 | 56 | 2.79*3.41 | 106 | 2.36*4.46 | 156 | 1.38*7.57 |
| 7 | 1.34*7.18 | 57 | 1.19*8.12 | 107 | 2.44*4.29 | 157 | 2.19*4.82 |
| 8 | 2.67*3.57 | 58 | 1.47*6.53 | 108 | 1.34*7.83 | 158 | 2.45*4.31 |
| 9 | 1.21*8.13 | 59 | 2.85*3.35 | 109 | 1.61*6.52 | 159 | 1.39*7.54 |
| 10 | 2.18*4.34 | 60 | 2.65*3.59 | 110 | 2.66*3.93 | 160 | 1.43*7.34 |
| 11 | 2.56*3.71 | 61 | 1.94*4.89 | 111 | 1.57*6.69 | 161 | 2.91*3.62 |
| 12 | 1.49*6.43 | 62 | 1.36*7.14 | 112 | 1.71*6.17 | 162 | 2.79*3.76 |
| 13 | 1.77*5.36 | 63 | 1.72*5.53 | 113 | 2.59*4.11 | 163 | 2.15*4.85 |
| 14 | 2.92*3.26 | 64 | 2.76*3.46 | 114 | 1.53*6.87 | 164 | 1.79*5.84 |
| 15 | 1.53*6.24 | 65 | 2.77*3.44 | 115 | 1.22*8.57 | 165 | 1.56*6.73 |
| 16 | 2.51*3.79 | 66 | 2.72*3.49 | 116 | 1.33*7.86 | 166 | 3.11*3.38 |
| 17 | 2.13*4.45 | 67 | 1.23*7.84 | 117 | 2.16*4.86 | 167 | 1.62*6.47 |
| 18 | 2.75*3.45 | 68 | 2.35*4.11 | 118 | 2.54*4.16 | 168 | 2.76*3.81 |
| 19 | 2.93*3.25 | 69 | 2.88*3.31 | 119 | 1.55*6.79 | 169 | 2.83*3.72 |
| 20 | 2.39*3.95 | 70 | 2.27*4.19 | 120 | 3.19*3.28 | 170 | 2.43*4.32 |
| 21 | 1.64*5.79 | 71 | 2.21*4.33 | 121 | 1.37*7.63 | 171 | 2.34*4.49 |
| 22 | 1.25*7.75 | 72 | 1.58*5.97 | 122 | 2.32*4.52 | 172 | 1.76*5.92 |
| 23 | 2.59*3.67 | 73 | 1.91*5.11 | 123 | 1.94*5.39 | 173 | 2.52*4.18 |
| 24 | 1.13*8.32 | 74 | 2.69*3.55 | 124 | 2.72*3.87 | 174 | 1.92*5.48 |
| 25 | 1.56*6.14 | 75 | 1.59*5.96 | 125 | 1.52*6.92 | 175 | 1.16*8.93 |
| 26 | 1.59*6.11 | 76 | 2.33*4.14 | 126 | 1.42*7.38 | 176 | 2.97*3.53 |
| 27 | 1.17*8.18 | 77 | 1.65*5.75 | 127 | 3.15*3.33 | 177 | 2.38*4.42 |
| 28 | 1.55*6.15 | 78 | 2.22*4.32 | 128 | 1.93*5.43 | 178 | 3.23*3.25 |
| 29 | 1.33*7.12 | 79 | 1.83*5.24 | 129 | 1.49*6.95 | 179 | 1.31*7.96 |
| 30 | 1.54*6.19 | 80 | 1.19*7.95 | 130 | 2.51*4.22 | 180 | 1.83*5.75 |
| 31 | 1.92*4.92 | 81 | 1.62*5.89 | 131 | 2.18*4.83 | 181 | 2.74*3.84 |
| 32 | 1.43*6.65 | 82 | 1.48*6.47 | 132 | 2.94*3.58 | 182 | 1.75*5.95 |
| 33 | 1.79*5.29 | 83 | 2.66*3.58 | 133 | 2.82*3.73 | 183 | 2.39*4.39 |
| 34 | 2.73*3.47 | 84 | 1.58*6.12 | 134 | 1.66*6.32 | 184 | 1.81*5.83 |
| 35 | 2.71*3.51 | 85 | 2.17*4.37 | 135 | 2.64*3.94 | 185 | 1.74*6.13 |
| 36 | 1.66*5.69 | 86 | 2.55*3.73 | 136 | 1.49*7.11 | 186 | 3.16*3.32 |
| 37 | 1.11*8.58 | 87 | 1.22*8.11 | 137 | 1.44*7.26 | 187 | 2.26*4.63 |
| 38 | 2.25*4.23 | 88 | 1.88*5.12 | 138 | 2.75*3.83 | 188 | 1.17*8.91 |
| 39 | 2.83*3.36 | 89 | 2.53*3.75 | 139 | 2.93*3.59 | 189 | 2.57*4.13 |
| 40 | 2.91*3.27 | 90 | 1.89*5.11 | 140 | 2.89*3.63 | 190 | 2.41*4.36 |
| 41 | 2.86*3.33 | 91 | 2.96*3.22 | 141 | 2.81*3.74 | 191 | 1.86*5.63 |
| 42 | 1.18*8.13 | 92 | 2.48*3.84 | 142 | 1.85*5.65 | 192 | 2.63*3.96 |
| 43 | 1.15*8.23 | 93 | 1.44*6.63 | 143 | 2.47*4.25 | 193 | 2.95*3.57 |
| 44 | 2.82*3.37 | 94 | 1.91*4.93 | 144 | 2.92*3.61 | 194 | 2.24*4.68 |
| 45 | 2.42*3.92 | 95 | 1.22*7.92 | 145 | 1.73*6.14 | 195 | 2.11*4.94 |
| 46 | 1.84*5.21 | 96 | 2.52*3.77 | 146 | 1.82*5.79 | 196 | 1.25*8.37 |
| 47 | 1.24*7.81 | 97 | 2.47*3.85 | 147 | 1.41*7.43 | 197 | 2.77*3.79 |
| 48 | 1.61*5.92 | 98 | 2.57*3.69 | 148 | 2.13*4.89 | 198 | 1.45*7.23 |
| 49 | 2.24*4.26 | 99 | 1.76*5.38 | 149 | 2.78*3.77 | 199 | 2.84*3.71 |
| 50 | 1.42*6.67 | 100 | 2.89*3.29 | 150 | 2.67*3.92 | 200 | 1.59*6.57 |
