# Supplementary material for: Effects of acute stress on biological motion perception
Source: PLoS One. 2024 Sep 18;19(9):e0310502. doi: 10.1371/journal.pone.0310502 (PMC11410201; doi:10.1371/journal.pone.0310502)
Supplement: S2 Appendix — (DOCX) [file pone.0310502.s002.docx]

**Table1 Key/answer mapping analysis for RT (Unit: ms)**

|  | Stress | Control |
| --- | --- | --- |
| Global upright | 730.29 | 812.06 |
| Global inverted | 989.84 | 1116.09 |
| Local upright | 777.53 | 877.54 |
| Local inverted | 963.91 | 1089.29 |
| Global | 860.06 | 964.08 |
| Local | 870.72 | 983.42 |
| Upright | 753.91 | 844.8 |
| Inverted | 976.87 | 1102.69 |

| **Tests of Within-Subjects Effects** | | | | | | | |
| --- | --- | --- | --- | --- | --- | --- | --- |
| Measure:MEASURE_1 | | | | | | | |
| Source | | Type III Sum of Squares | df | Mean Square | F | Sig. | Partial Eta Squared |
| stress level | Sphericity Assumed | 563571.692 | 1 | 563571.692 | 9.777 | .005 | .298 |
|  | Greenhouse-Geisser | 563571.692 | 1.000 | 563571.692 | 9.777 | .005 | .298 |
|  | Huynh-Feldt | 563571.692 | 1.000 | 563571.692 | 9.777 | .005 | .298 |
|  | Lower-bound | 563571.692 | 1.000 | 563571.692 | 9.777 | .005 | .298 |
| Error(stress level) | Sphericity Assumed | 1325793.977 | 23 | 57643.216 |  |  |  |
|  | Greenhouse-Geisser | 1325793.977 | 23.000 | 57643.216 |  |  |  |
|  | Huynh-Feldt | 1325793.977 | 23.000 | 57643.216 |  |  |  |
|  | Lower-bound | 1325793.977 | 23.000 | 57643.216 |  |  |  |
| contour characteristic | Sphericity Assumed | 10796.400 | 1 | 10796.400 | 1.358 | .256 | .056 |
|  | Greenhouse-Geisser | 10796.400 | 1.000 | 10796.400 | 1.358 | .256 | .056 |
|  | Huynh-Feldt | 10796.400 | 1.000 | 10796.400 | 1.358 | .256 | .056 |
|  | Lower-bound | 10796.400 | 1.000 | 10796.400 | 1.358 | .256 | .056 |
| Error(contour characteristic) | Sphericity Assumed | 182841.913 | 23 | 7949.648 |  |  |  |
|  | Greenhouse-Geisser | 182841.913 | 23.000 | 7949.648 |  |  |  |
|  | Huynh-Feldt | 182841.913 | 23.000 | 7949.648 |  |  |  |
|  | Lower-bound | 182841.913 | 23.000 | 7949.648 |  |  |  |
| motion characteristic | Sphericity Assumed | 2774624.713 | 1 | 2774624.713 | 161.487 | .000 | .875 |
|  | Greenhouse-Geisser | 2774624.713 | 1.000 | 2774624.713 | 161.487 | .000 | .875 |
|  | Huynh-Feldt | 2774624.713 | 1.000 | 2774624.713 | 161.487 | .000 | .875 |
|  | Lower-bound | 2774624.713 | 1.000 | 2774624.713 | 161.487 | .000 | .875 |
| Error(motion characteristic) | Sphericity Assumed | 395178.502 | 23 | 17181.674 |  |  |  |
|  | Greenhouse-Geisser | 395178.502 | 23.000 | 17181.674 |  |  |  |
|  | Huynh-Feldt | 395178.502 | 23.000 | 17181.674 |  |  |  |
|  | Lower-bound | 395178.502 | 23.000 | 17181.674 |  |  |  |
| stress level * contour characteristic | Sphericity Assumed | 903.501 | 1 | 903.501 | .769 | .389 | .032 |
|  | Greenhouse-Geisser | 903.501 | 1.000 | 903.501 | .769 | .389 | .032 |
|  | Huynh-Feldt | 903.501 | 1.000 | 903.501 | .769 | .389 | .032 |
|  | Lower-bound | 903.501 | 1.000 | 903.501 | .769 | .389 | .032 |
| Error(stress level*contour characteristic) | Sphericity Assumed | 27009.915 | 23 | 1174.344 |  |  |  |
|  | Greenhouse-Geisser | 27009.915 | 23.000 | 1174.344 |  |  |  |
|  | Huynh-Feldt | 27009.915 | 23.000 | 1174.344 |  |  |  |
|  | Lower-bound | 27009.915 | 23.000 | 1174.344 |  |  |  |
| stress level * motion characteristic | Sphericity Assumed | 14641.259 | 1 | 14641.259 | 3.520 | .073 | .133 |
|  | Greenhouse-Geisser | 14641.259 | 1.000 | 14641.259 | 3.520 | .073 | .133 |
|  | Huynh-Feldt | 14641.259 | 1.000 | 14641.259 | 3.520 | .073 | .133 |
|  | Lower-bound | 14641.259 | 1.000 | 14641.259 | 3.520 | .073 | .133 |
| Error(stress level*motion characteristic) | Sphericity Assumed | 95655.317 | 23 | 4158.927 |  |  |  |
|  | Greenhouse-Geisser | 95655.317 | 23.000 | 4158.927 |  |  |  |
|  | Huynh-Feldt | 95655.317 | 23.000 | 4158.927 |  |  |  |
|  | Lower-bound | 95655.317 | 23.000 | 4158.927 |  |  |  |
| contour characteristic * motion characteristic | Sphericity Assumed | 82126.071 | 1 | 82126.071 | 17.474 | .000 | .432 |
|  | Greenhouse-Geisser | 82126.071 | 1.000 | 82126.071 | 17.474 | .000 | .432 |
|  | Huynh-Feldt | 82126.071 | 1.000 | 82126.071 | 17.474 | .000 | .432 |
|  | Lower-bound | 82126.071 | 1.000 | 82126.071 | 17.474 | .000 | .432 |
| Error(contour characteristic*motion characteristic) | Sphericity Assumed | 108100.557 | 23 | 4700.024 |  |  |  |
|  | Greenhouse-Geisser | 108100.557 | 23.000 | 4700.024 |  |  |  |
|  | Huynh-Feldt | 108100.557 | 23.000 | 4700.024 |  |  |  |
|  | Lower-bound | 108100.557 | 23.000 | 4700.024 |  |  |  |
| stress level * contour characteristic * motion characteristic | Sphericity Assumed | 1095.099 | 1 | 1095.099 | .644 | .430 | .027 |
|  | Greenhouse-Geisser | 1095.099 | 1.000 | 1095.099 | .644 | .430 | .027 |
|  | Huynh-Feldt | 1095.099 | 1.000 | 1095.099 | .644 | .430 | .027 |
|  | Lower-bound | 1095.099 | 1.000 | 1095.099 | .644 | .430 | .027 |
| Error(stress level*contour characteristic*motion characteristic) | Sphericity Assumed | 39097.362 | 23 | 1699.885 |  |  |  |
|  | Greenhouse-Geisser | 39097.362 | 23.000 | 1699.885 |  |  |  |
|  | Huynh-Feldt | 39097.362 | 23.000 | 1699.885 |  |  |  |
|  | Lower-bound | 39097.362 | 23.000 | 1699.885 |  |  |  |

**Table2 Key/answer mapping analysis for accuracy**

|  | Stress | Control |
| --- | --- | --- |
| Global upright | 0.98 | 0.99 |
| Global inverted | 0.91 | 0.89 |
| Local upright | 0.97 | 0.98 |
| Local inverted | 0.83 | 0.86 |
| Global | 0.95 | 0.94 |
| Local | 0.9 | 0.92 |
| Upright | 0.97 | 0.99 |
| Inverted | 0.87 | 0.88 |

| **stress level** | | | | |
| --- | --- | --- | --- | --- |
|  | Observed N | | Expected N | Residual |
| Stress | 4417 | | 4442.5 | -25.5 |
| Control | 4468 | | 4442.5 | 25.5 |
| Total | 8885 | |  |  |
| **Test Statistics** | | | | |
|  | | stress level | | |
| Chi-Square | | .293^a^ | | |
| df | | 1 | | |
| Asymp. Sig. | | .588 | | |
| a. 0 cells (.0%) have expected frequencies less than 5. The minimum expected cell frequency is 4442.5. | | | | |
| **motion characteristic** | | | | |
|  | Observed N | | Expected N | Residual |
| Upright | 4700 | | 4442.5 | 257.5 |
| Inverted | 4185 | | 4442.5 | -257.5 |
| Total | 8885 | |  |  |
| **Test Statistics** | | | | |
|  | | motion characteristic | | |
| Chi-Square | | 29.851^a^ | | |
| df | | 1 | | |
| Asymp. Sig. | | .000 | | |
| a. 0 cells (.0%) have expected frequencies less than 5. The minimum expected cell frequency is 4442.5. | | | | |
| **contour characteristic** | | | | |
|  | Observed N | | Expected N | Residual |
| Global | 4525 | | 4442.5 | 82.5 |
| Local | 4360 | | 4442.5 | -82.5 |
| Total | 8885 | |  |  |
| **Test Statistics** | | | | |
|  | | contour characteristic | | |
| Chi-Square | | 3.064^a^ | | |
| df | | 1 | | |
| Asymp. Sig. | | .080 | | |
| a. 0 cells (.0%) have expected frequencies less than 5. The minimum expected cell frequency is 4442.5. | | | | |
