# Supplementary material for: Effects of acute stress on biological motion perception
Source: PLoS One. 2024 Sep 18;19(9):e0310502. doi: 10.1371/journal.pone.0310502 (PMC11410201; doi:10.1371/journal.pone.0310502)
Supplement: S3 Appendix — (DOCX) [file pone.0310502.s003.docx]

**Table1 Key/answer mapping analysis for P1 peak latency**

| **Tests of Within-Subjects Effects** | | | | | | | |
| --- | --- | --- | --- | --- | --- | --- | --- |
| Measure:MEASURE_1 | | | | | | | |
| Source | | Type III Sum of Squares | df | Mean Square | F | Sig. | Partial Eta Squared |
| electrode site | Sphericity Assumed | 10191.881 | 4 | 2547.970 | 7.387 | .000 | .243 |
|  | Greenhouse-Geisser | 10191.881 | 2.945 | 3460.158 | 7.387 | .000 | .243 |
| stress level | Sphericity Assumed | 430.676 | 1 | 430.676 | 3.132 | .090 | .120 |
|  | Greenhouse-Geisser | 430.676 | 1.000 | 430.676 | 3.132 | .090 | .120 |
| contour characteristic | Sphericity Assumed | 48.151 | 1 | 48.151 | .185 | .671 | .008 |
|  | Greenhouse-Geisser | 48.151 | 1.000 | 48.151 | .185 | .671 | .008 |
| motion characteristic | Sphericity Assumed | 1552.959 | 1 | 1552.959 | 8.655 | .007 | .273 |
|  | Greenhouse-Geisser | 1552.959 | 1.000 | 1552.959 | 8.655 | .007 | .273 |
| electrode site * stress level | Sphericity Assumed | 149.173 | 4 | 37.293 | .553 | .697 | .023 |
|  | Greenhouse-Geisser | 149.173 | 2.865 | 52.076 | .553 | .640 | .023 |
| electrode site * contour characteristic | Sphericity Assumed | 150.802 | 4 | 37.701 | .407 | .803 | .017 |
|  | Greenhouse-Geisser | 150.802 | 3.049 | 49.459 | .407 | .752 | .017 |
| stress level * contour characteristic | Sphericity Assumed | 33.376 | 1 | 33.376 | .301 | .589 | .013 |
|  | Greenhouse-Geisser | 33.376 | 1.000 | 33.376 | .301 | .589 | .013 |
| electrode site * stress level * contour characteristic | Sphericity Assumed | 139.577 | 4 | 34.894 | .665 | .618 | .028 |
|  | Greenhouse-Geisser | 139.577 | 3.075 | 45.396 | .665 | .580 | .028 |
| electrode site * motion characteristic | Sphericity Assumed | 287.702 | 4 | 71.926 | .825 | .513 | .035 |
|  | Greenhouse-Geisser | 287.702 | 3.002 | 95.841 | .825 | .485 | .035 |
| stress level * motion characteristic | Sphericity Assumed | 184.626 | 1 | 184.626 | 1.601 | .218 | .065 |
|  | Greenhouse-Geisser | 184.626 | 1.000 | 184.626 | 1.601 | .218 | .065 |
| electrode site * stress level * motion characteristic | Sphericity Assumed | 105.077 | 4 | 26.269 | .405 | .805 | .017 |
|  | Greenhouse-Geisser | 105.077 | 3.254 | 32.296 | .405 | .766 | .017 |
| contour characteristic * motion characteristic | Sphericity Assumed | 2428.884 | 1 | 2428.884 | 7.927 | .010 | .256 |
|  | Greenhouse-Geisser | 2428.884 | 1.000 | 2428.884 | 7.927 | .010 | .256 |
| electrode site * contour characteristic * motion characteristic | Sphericity Assumed | 867.173 | 4 | 216.793 | 3.398 | .012 | .129 |
|  | Greenhouse-Geisser | 867.173 | 3.151 | 275.241 | 3.398 | .021 | .129 |
| stress level * contour characteristic * motion characteristic | Sphericity Assumed | 5.859 | 1 | 5.859 | .029 | .867 | .001 |
|  | Greenhouse-Geisser | 5.859 | 1.000 | 5.859 | .029 | .867 | .001 |
| electrode site * stress level * contour characteristic * motion characteristic | Sphericity Assumed | 70.865 | 4 | 17.716 | .238 | .916 | .010 |
|  | Greenhouse-Geisser | 70.865 | 2.987 | 23.723 | .238 | .869 | .010 |

**Table2 Key/answer mapping analysis for P1 peak amplitude**

| **Tests of Within-Subjects Effects** | | | | | | | |
| --- | --- | --- | --- | --- | --- | --- | --- |
| Measure:MEASURE_1 | | | | | | | |
| Source | | Type III Sum of Squares | df | Mean Square | F | Sig. | Partial Eta Squared |
| electrode site | Sphericity Assumed | 37.948 | 4 | 9.487 | .460 | .765 | .020 |
|  | Greenhouse-Geisser | 37.948 | 2.463 | 15.405 | .460 | .674 | .020 |
| stress level | Sphericity Assumed | 12.304 | 1 | 12.304 | .527 | .475 | .022 |
|  | Greenhouse-Geisser | 12.304 | 1.000 | 12.304 | .527 | .475 | .022 |
| contour characteristic | Sphericity Assumed | 41.789 | 1 | 41.789 | 4.935 | .036 | .177 |
|  | Greenhouse-Geisser | 41.789 | 1.000 | 41.789 | 4.935 | .036 | .177 |
| motion characteristic | Sphericity Assumed | 11.922 | 1 | 11.922 | 1.751 | .199 | .071 |
|  | Greenhouse-Geisser | 11.922 | 1.000 | 11.922 | 1.751 | .199 | .071 |
| electrode site * stress level | Sphericity Assumed | 17.041 | 4 | 4.260 | 2.578 | .043 | .101 |
|  | Greenhouse-Geisser | 17.041 | 2.230 | 7.642 | 2.578 | .080 | .101 |
| electrode site * contour characteristic | Sphericity Assumed | 4.062 | 4 | 1.016 | 1.191 | .320 | .049 |
|  | Greenhouse-Geisser | 4.062 | 2.928 | 1.387 | 1.191 | .319 | .049 |
| stress level * contour characteristic | Sphericity Assumed | 4.711 | 1 | 4.711 | .897 | .353 | .038 |
|  | Greenhouse-Geisser | 4.711 | 1.000 | 4.711 | .897 | .353 | .038 |
| electrode site * stress level * contour characteristic | Sphericity Assumed | 1.555 | 4 | .389 | .602 | .662 | .025 |
|  | Greenhouse-Geisser | 1.555 | 2.631 | .591 | .602 | .595 | .025 |
| electrode site * motion characteristic | Sphericity Assumed | 7.695 | 4 | 1.924 | 1.936 | .111 | .078 |
|  | Greenhouse-Geisser | 7.695 | 2.949 | 2.610 | 1.936 | .133 | .078 |
| stress level * motion characteristic | Sphericity Assumed | .166 | 1 | .166 | .025 | .875 | .001 |
|  | Greenhouse-Geisser | .166 | 1.000 | .166 | .025 | .875 | .001 |
| electrode site * stress level * motion characteristic | Sphericity Assumed | 4.125 | 4 | 1.031 | 1.096 | .363 | .045 |
|  | Greenhouse-Geisser | 4.125 | 2.429 | 1.698 | 1.096 | .350 | .045 |
| contour characteristic * motion characteristic | Sphericity Assumed | 113.680 | 1 | 113.680 | 12.669 | .002 | .355 |
|  | Greenhouse-Geisser | 113.680 | 1.000 | 113.680 | 12.669 | .002 | .355 |
| electrode site * contour characteristic * motion characteristic | Sphericity Assumed | 3.054 | 4 | .763 | .930 | .450 | .039 |
|  | Greenhouse-Geisser | 3.054 | 2.724 | 1.121 | .930 | .424 | .039 |
| stress level * contour characteristic * motion characteristic | Sphericity Assumed | 1.087 | 1 | 1.087 | .191 | .666 | .008 |
|  | Greenhouse-Geisser | 1.087 | 1.000 | 1.087 | .191 | .666 | .008 |
| electrode site * stress level * contour characteristic * motion characteristic | Sphericity Assumed | 2.090 | 4 | .522 | .576 | .681 | .024 |
|  | Greenhouse-Geisser | 2.090 | 2.395 | .872 | .576 | .596 | .024 |

**Table3 Key/answer mapping analysis for P2 peak latency**

| **Tests of Within-Subjects Effects** | | | | | | | |
| --- | --- | --- | --- | --- | --- | --- | --- |
| Measure:MEASURE_1 | | | | | | | |
| Source | | Type III Sum of Squares | df | Mean Square | F | Sig. | Partial Eta Squared |
| electrode site | Sphericity Assumed | 2996.077 | 3 | 998.692 | .873 | .460 | .037 |
|  | Greenhouse-Geisser | 2996.077 | 1.222 | 2451.982 | .873 | .379 | .037 |
| stress level | Sphericity Assumed | 4223.439 | 1 | 4223.439 | 2.721 | .113 | .106 |
|  | Greenhouse-Geisser | 4223.439 | 1.000 | 4223.439 | 2.721 | .113 | .106 |
| motion characteristic | Sphericity Assumed | 5606.283 | 1 | 5606.283 | 3.019 | .096 | .116 |
|  | Greenhouse-Geisser | 5606.283 | 1.000 | 5606.283 | 3.019 | .096 | .116 |
| contour characteristic | Sphericity Assumed | 24899.908 | 1 | 24899.908 | 23.067 | .000 | .501 |
|  | Greenhouse-Geisser | 24899.908 | 1.000 | 24899.908 | 23.067 | .000 | .501 |
| electrode site * stress level | Sphericity Assumed | 840.077 | 3 | 280.026 | .855 | .469 | .036 |
|  | Greenhouse-Geisser | 840.077 | 1.610 | 521.945 | .855 | .412 | .036 |
| electrode site * motion characteristic | Sphericity Assumed | 4072.212 | 3 | 1357.404 | 2.768 | .048 | .107 |
|  | Greenhouse-Geisser | 4072.212 | 1.552 | 2623.320 | 2.768 | .088 | .107 |
| stress level * motion characteristic | Sphericity Assumed | 3557.824 | 1 | 3557.824 | 3.246 | .085 | .124 |
|  | Greenhouse-Geisser | 3557.824 | 1.000 | 3557.824 | 3.246 | .085 | .124 |
| electrode site * stress level * motion characteristic | Sphericity Assumed | 2827.129 | 3 | 942.376 | 2.504 | .066 | .098 |
|  | Greenhouse-Geisser | 2827.129 | 1.735 | 1629.685 | 2.504 | .101 | .098 |
| electrode site * contour characteristic | Sphericity Assumed | 599.796 | 3 | 199.932 | .494 | .688 | .021 |
|  | Greenhouse-Geisser | 599.796 | 1.360 | 441.069 | .494 | .544 | .021 |
| stress level * contour characteristic | Sphericity Assumed | 51.564 | 1 | 51.564 | .053 | .820 | .002 |
|  | Greenhouse-Geisser | 51.564 | 1.000 | 51.564 | .053 | .820 | .002 |
| electrode site * stress level * contour characteristic | Sphericity Assumed | 388.098 | 3 | 129.366 | .429 | .733 | .018 |
|  | Greenhouse-Geisser | 388.098 | 2.071 | 187.355 | .429 | .660 | .018 |
| motion characteristic * contour characteristic | Sphericity Assumed | 15597.033 | 1 | 15597.033 | 12.825 | .002 | .358 |
|  | Greenhouse-Geisser | 15597.033 | 1.000 | 15597.033 | 12.825 | .002 | .358 |
| electrode site * motion characteristic * contour characteristic | Sphericity Assumed | 426.316 | 3 | 142.105 | .278 | .841 | .012 |
|  | Greenhouse-Geisser | 426.316 | 1.314 | 324.505 | .278 | .665 | .012 |
| stress level * motion characteristic * contour characteristic | Sphericity Assumed | 23.730 | 1 | 23.730 | .014 | .907 | .001 |
|  | Greenhouse-Geisser | 23.730 | 1.000 | 23.730 | .014 | .907 | .001 |
| electrode site * stress level * motion characteristic * contour characteristic | Sphericity Assumed | 798.035 | 3 | 266.012 | .615 | .608 | .026 |
|  | Greenhouse-Geisser | 798.035 | 1.519 | 525.405 | .615 | .503 | .026 |

**Table4 Key/answer mapping analysis for P2 peak amplitude**

| **Tests of Within-Subjects Effects** | | | | | | | |
| --- | --- | --- | --- | --- | --- | --- | --- |
| Measure:MEASURE_1 | | | | | | | |
| Source | | Type III Sum of Squares | df | Mean Square | F | Sig. | Partial Eta Squared |
| electrode site | Sphericity Assumed | 343.788 | 3 | 114.596 | 3.821 | .014 | .142 |
|  | Greenhouse-Geisser | 343.788 | 1.511 | 227.476 | 3.821 | .043 | .142 |
| stress level | Sphericity Assumed | 385.711 | 1 | 385.711 | 8.839 | .007 | .278 |
|  | Greenhouse-Geisser | 385.711 | 1.000 | 385.711 | 8.839 | .007 | .278 |
| motion characteristic | Sphericity Assumed | 3.754 | 1 | 3.754 | .403 | .532 | .017 |
|  | Greenhouse-Geisser | 3.754 | 1.000 | 3.754 | .403 | .532 | .017 |
| contour characteristic | Sphericity Assumed | 359.428 | 1 | 359.428 | 22.235 | .000 | .492 |
|  | Greenhouse-Geisser | 359.428 | 1.000 | 359.428 | 22.235 | .000 | .492 |
| electrode site * stress level | Sphericity Assumed | 1.784 | 3 | .595 | .211 | .888 | .009 |
|  | Greenhouse-Geisser | 1.784 | 1.579 | 1.130 | .211 | .758 | .009 |
| electrode site * motion characteristic | Sphericity Assumed | 39.037 | 3 | 13.012 | 10.306 | .000 | .309 |
|  | Greenhouse-Geisser | 39.037 | 2.151 | 18.146 | 10.306 | .000 | .309 |
| stress level * motion characteristic | Sphericity Assumed | 3.583 | 1 | 3.583 | .778 | .387 | .033 |
|  | Greenhouse-Geisser | 3.583 | 1.000 | 3.583 | .778 | .387 | .033 |
| electrode site * stress level * motion characteristic | Sphericity Assumed | .769 | 3 | .256 | .279 | .840 | .012 |
|  | Greenhouse-Geisser | .769 | 1.704 | .451 | .279 | .723 | .012 |
| electrode site * contour characteristic | Sphericity Assumed | 8.905 | 3 | 2.968 | 1.531 | .214 | .062 |
|  | Greenhouse-Geisser | 8.905 | 1.493 | 5.962 | 1.531 | .231 | .062 |
| stress level * contour characteristic | Sphericity Assumed | .099 | 1 | .099 | .016 | .899 | .001 |
|  | Greenhouse-Geisser | .099 | 1.000 | .099 | .016 | .899 | .001 |
| electrode site * stress level * contour characteristic | Sphericity Assumed | 7.552 | 3 | 2.517 | 3.397 | .023 | .129 |
|  | Greenhouse-Geisser | 7.552 | 1.580 | 4.781 | 3.397 | .055 | .129 |
| motion characteristic * contour characteristic | Sphericity Assumed | 1148.927 | 1 | 1148.927 | 82.964 | .000 | .783 |
|  | Greenhouse-Geisser | 1148.927 | 1.000 | 1148.927 | 82.964 | .000 | .783 |
| electrode site * motion characteristic * contour characteristic | Sphericity Assumed | 29.415 | 3 | 9.805 | 5.984 | .001 | .206 |
|  | Greenhouse-Geisser | 29.415 | 2.232 | 13.176 | 5.984 | .003 | .206 |
| stress level * motion characteristic * contour characteristic | Sphericity Assumed | .764 | 1 | .764 | .112 | .741 | .005 |
|  | Greenhouse-Geisser | .764 | 1.000 | .764 | .112 | .741 | .005 |
| electrode site * stress level * motion characteristic * contour characteristic | Sphericity Assumed | .191 | 3 | .064 | .073 | .974 | .003 |
|  | Greenhouse-Geisser | .191 | 1.671 | .114 | .073 | .900 | .003 |

**Table5 Key/answer mapping analysis for N330 peak latency**

| **Tests of Within-Subjects Effects** | | | | | | | |
| --- | --- | --- | --- | --- | --- | --- | --- |
| Measure:MEASURE_1 | | | | | | | |
| Source | | Type III Sum of Squares | df | Mean Square | F | Sig. | Partial Eta Squared |
| electrode site | Sphericity Assumed | 80.667 | 1 | 80.667 | .842 | .368 | .035 |
|  | Greenhouse-Geisser | 80.667 | 1.000 | 80.667 | .842 | .368 | .035 |
| stress level | Sphericity Assumed | 135.375 | 1 | 135.375 | .095 | .760 | .004 |
|  | Greenhouse-Geisser | 135.375 | 1.000 | 135.375 | .095 | .760 | .004 |
| contour characteristic | Sphericity Assumed | 38680.510 | 1 | 38680.510 | 25.951 | .000 | .530 |
|  | Greenhouse-Geisser | 38680.510 | 1.000 | 38680.510 | 25.951 | .000 | .530 |
| motion characteristic | Sphericity Assumed | 62373.010 | 1 | 62373.010 | 27.759 | .000 | .547 |
|  | Greenhouse-Geisser | 62373.010 | 1.000 | 62373.010 | 27.759 | .000 | .547 |
| electrode site * stress level | Sphericity Assumed | 142.594 | 1 | 142.594 | 1.747 | .199 | .071 |
|  | Greenhouse-Geisser | 142.594 | 1.000 | 142.594 | 1.747 | .199 | .071 |
| electrode site * contour characteristic | Sphericity Assumed | 376.042 | 1 | 376.042 | 5.232 | .032 | .185 |
|  | Greenhouse-Geisser | 376.042 | 1.000 | 376.042 | 5.232 | .032 | .185 |
| stress level * contour characteristic | Sphericity Assumed | 1.042 | 1 | 1.042 | .001 | .976 | .000 |
|  | Greenhouse-Geisser | 1.042 | 1.000 | 1.042 | .001 | .976 | .000 |
| electrode site * stress level * contour characteristic | Sphericity Assumed | 75.260 | 1 | 75.260 | .936 | .343 | .039 |
|  | Greenhouse-Geisser | 75.260 | 1.000 | 75.260 | .936 | .343 | .039 |
| electrode site * motion characteristic | Sphericity Assumed | 9.375 | 1 | 9.375 | .122 | .730 | .005 |
|  | Greenhouse-Geisser | 9.375 | 1.000 | 9.375 | .122 | .730 | .005 |
| stress level * motion characteristic | Sphericity Assumed | 1426.042 | 1 | 1426.042 | 1.394 | .250 | .057 |
|  | Greenhouse-Geisser | 1426.042 | 1.000 | 1426.042 | 1.394 | .250 | .057 |
| electrode site * stress level * motion characteristic | Sphericity Assumed | 119.260 | 1 | 119.260 | 1.832 | .189 | .074 |
|  | Greenhouse-Geisser | 119.260 | 1.000 | 119.260 | 1.832 | .189 | .074 |
| contour characteristic * motion characteristic | Sphericity Assumed | 42883.760 | 1 | 42883.760 | 29.201 | .000 | .559 |
|  | Greenhouse-Geisser | 42883.760 | 1.000 | 42883.760 | 29.201 | .000 | .559 |
| electrode site * contour characteristic * motion characteristic | Sphericity Assumed | 24.000 | 1 | 24.000 | .306 | .585 | .013 |
|  | Greenhouse-Geisser | 24.000 | 1.000 | 24.000 | .306 | .585 | .013 |
| stress level * contour characteristic * motion characteristic | Sphericity Assumed | 376.042 | 1 | 376.042 | .243 | .627 | .010 |
|  | Greenhouse-Geisser | 376.042 | 1.000 | 376.042 | .243 | .627 | .010 |
| electrode site * stress level * contour characteristic * motion characteristic | Sphericity Assumed | 133.010 | 1 | 133.010 | 1.562 | .224 | .064 |
|  | Greenhouse-Geisser | 133.010 | 1.000 | 133.010 | 1.562 | .224 | .064 |

**Table6 Key/answer mapping analysis for N330 peak amplitude**

| **Tests of Within-Subjects Effects** | | | | | | | |
| --- | --- | --- | --- | --- | --- | --- | --- |
| Measure:MEASURE_1 | | | | | | | |
| Source | | Type III Sum of Squares | df | Mean Square | F | Sig. | Partial Eta Squared |
| electrode site | Sphericity Assumed | 24.399 | 1 | 24.399 | .542 | .469 | .023 |
|  | Greenhouse-Geisser | 24.399 | 1.000 | 24.399 | .542 | .469 | .023 |
| stress level | Sphericity Assumed | 564.631 | 1 | 564.631 | 9.351 | .006 | .289 |
|  | Greenhouse-Geisser | 564.631 | 1.000 | 564.631 | 9.351 | .006 | .289 |
| contour characteristic | Sphericity Assumed | 29.746 | 1 | 29.746 | .171 | .683 | .007 |
|  | Greenhouse-Geisser | 29.746 | 1.000 | 29.746 | .171 | .683 | .007 |
| motion characteristic | Sphericity Assumed | 447.475 | 1 | 447.475 | 2.971 | .098 | .114 |
|  | Greenhouse-Geisser | 447.475 | 1.000 | 447.475 | 2.971 | .098 | .114 |
| electrode site * stress level | Sphericity Assumed | 4.024 | 1 | 4.024 | 1.226 | .280 | .051 |
|  | Greenhouse-Geisser | 4.024 | 1.000 | 4.024 | 1.226 | .280 | .051 |
| electrode site * contour characteristic | Sphericity Assumed | 40.058 | 1 | 40.058 | 1.619 | .216 | .066 |
|  | Greenhouse-Geisser | 40.058 | 1.000 | 40.058 | 1.619 | .216 | .066 |
| stress level * contour characteristic | Sphericity Assumed | 76.235 | 1 | 76.235 | .761 | .392 | .032 |
|  | Greenhouse-Geisser | 76.235 | 1.000 | 76.235 | .761 | .392 | .032 |
| electrode site * stress level * contour characteristic | Sphericity Assumed | 13.821 | 1 | 13.821 | .777 | .387 | .033 |
|  | Greenhouse-Geisser | 13.821 | 1.000 | 13.821 | .777 | .387 | .033 |
| electrode site * motion characteristic | Sphericity Assumed | 13.308 | 1 | 13.308 | .682 | .418 | .029 |
|  | Greenhouse-Geisser | 13.308 | 1.000 | 13.308 | .682 | .418 | .029 |
| stress level * motion characteristic | Sphericity Assumed | 781.467 | 1 | 781.467 | 1.199 | .285 | .050 |
|  | Greenhouse-Geisser | 781.467 | 1.000 | 781.467 | 1.199 | .285 | .050 |
| electrode site * stress level * motion characteristic | Sphericity Assumed | 130.967 | 1 | 130.967 | .967 | .336 | .040 |
|  | Greenhouse-Geisser | 130.967 | 1.000 | 130.967 | .967 | .336 | .040 |
| contour characteristic * motion characteristic | Sphericity Assumed | 884.782 | 1 | 884.782 | 12.111 | .002 | .345 |
|  | Greenhouse-Geisser | 884.782 | 1.000 | 884.782 | 12.111 | .002 | .345 |
| electrode site * contour characteristic * motion characteristic | Sphericity Assumed | 23.030 | 1 | 23.030 | 1.137 | .297 | .047 |
|  | Greenhouse-Geisser | 23.030 | 1.000 | 23.030 | 1.137 | .297 | .047 |
| stress level * contour characteristic * motion characteristic | Sphericity Assumed | 47.145 | 1 | 47.145 | .594 | .449 | .025 |
|  | Greenhouse-Geisser | 47.145 | 1.000 | 47.145 | .594 | .449 | .025 |
| electrode site * stress level * contour characteristic * motion characteristic | Sphericity Assumed | 31.511 | 1 | 31.511 | 1.592 | .220 | .065 |
|  | Greenhouse-Geisser | 31.511 | 1.000 | 31.511 | 1.592 | .220 | .065 |

**Table7 Key/answer mapping analysis for LPP mean amplitude**

| **Tests of Within-Subjects Effects** | | | | | | | |
| --- | --- | --- | --- | --- | --- | --- | --- |
| Measure:MEASURE_1 | | | | | | | |
| Source | | Type III Sum of Squares | df | Mean Square | F | Sig. | Partial Eta Squared |
| electrode site | Sphericity Assumed | 49.610 | 2 | 24.805 | 7.456 | .002 | .245 |
|  | Greenhouse-Geisser | 49.610 | 1.730 | 28.683 | 7.456 | .003 | .245 |
| stress level | Sphericity Assumed | 696.035 | 1 | 696.035 | 13.678 | .001 | .373 |
|  | Greenhouse-Geisser | 696.035 | 1.000 | 696.035 | 13.678 | .001 | .373 |
|  | Sphericity Assumed | 696.035 | 1.000 | 696.035 | 13.678 | .001 | .373 |
|  | Greenhouse-Geisser | 696.035 | 1.000 | 696.035 | 13.678 | .001 | .373 |
| contour characteristic | Sphericity Assumed | 402.106 | 1 | 402.106 | 26.580 | .000 | .536 |
|  | Greenhouse-Geisser | 402.106 | 1.000 | 402.106 | 26.580 | .000 | .536 |
| motion characteristic | Sphericity Assumed | 104.463 | 1 | 104.463 | 4.595 | .043 | .167 |
|  | Greenhouse-Geisser | 104.463 | 1.000 | 104.463 | 4.595 | .043 | .167 |
| electrode site * stress level | Sphericity Assumed | .772 | 2 | .386 | .644 | .530 | .027 |
|  | Greenhouse-Geisser | .772 | 1.398 | .553 | .644 | .478 | .027 |
| electrode site * contour characteristic | Sphericity Assumed | 2.188 | 2 | 1.094 | 2.170 | .126 | .086 |
|  | Greenhouse-Geisser | 2.188 | 1.734 | 1.262 | 2.170 | .134 | .086 |
| stress level * contour characteristic | Sphericity Assumed | .176 | 1 | .176 | .024 | .879 | .001 |
|  | Greenhouse-Geisser | .176 | 1.000 | .176 | .024 | .879 | .001 |
| electrode site * stress level * contour characteristic | Sphericity Assumed | .013 | 2 | .007 | .021 | .979 | .001 |
|  | Greenhouse-Geisser | .013 | 1.718 | .008 | .021 | .967 | .001 |
| electrode site * motion characteristic | Sphericity Assumed | 10.976 | 2 | 5.488 | 7.048 | .002 | .235 |
|  | Greenhouse-Geisser | 10.976 | 1.584 | 6.928 | 7.048 | .005 | .235 |
| stress level * motion characteristic | Sphericity Assumed | 11.821 | 1 | 11.821 | 1.133 | .298 | .047 |
|  | Greenhouse-Geisser | 11.821 | 1.000 | 11.821 | 1.133 | .298 | .047 |
| electrode site * stress level * motion characteristic | Sphericity Assumed | .009 | 2 | .004 | .011 | .989 | .000 |
|  | Greenhouse-Geisser | .009 | 1.995 | .004 | .011 | .989 | .000 |
| contour characteristic * motion characteristic | Sphericity Assumed | 1455.458 | 1 | 1455.458 | 105.001 | .000 | .820 |
|  | Greenhouse-Geisser | 1455.458 | 1.000 | 1455.458 | 105.001 | .000 | .820 |
| electrode site * contour characteristic * motion characteristic | Sphericity Assumed | 2.204 | 2 | 1.102 | 2.813 | .070 | .109 |
|  | Greenhouse-Geisser | 2.204 | 1.965 | 1.121 | 2.813 | .071 | .109 |
| stress level * contour characteristic * motion characteristic | Sphericity Assumed | .498 | 1 | .498 | .059 | .810 | .003 |
|  | Greenhouse-Geisser | .498 | 1.000 | .498 | .059 | .810 | .003 |
| electrode site * stress level * contour characteristic * motion characteristic | Sphericity Assumed | .008 | 2 | .004 | .017 | .984 | .001 |
|  | Greenhouse-Geisser | .008 | 1.968 | .004 | .017 | .983 | .001 |
