# Supplementary material for: Effects of acute stress on biological motion perception
Source: PLoS One. 2024 Sep 18;19(9):e0310502. doi: 10.1371/journal.pone.0310502 (PMC11410201; doi:10.1371/journal.pone.0310502)
Supplement: Attachment — Submitted filename: Response to reviewers (new).docx [file pone.0310502.s004.docx]

**Response to Editor's Comments**

**Journal requirements:**

**Point 1:** Please ensure that your manuscript meets PLOS ONE's style requirements, including those for file naming.

**Response 1: Thanks for your suggestion. According to your suggestion, we have modified the manuscript to meet PLOS ONE’s style requirements.**

**Point 2:** Thank you for stating the following financial disclosure: [Humanities and Social Sciences Projects Funded by the Ministry of Education and the Scientific Research Project of Hubei Provincial Department of Education, China (Grant No. Q20221313)].

**Response 2: Thank you for the suggestion. According to your suggestion, we have modified in the section of “Funding information”. For details, see the red font on page 25, lines 854-859.**

**Point 3:** In the online submission form you indicate that your data is not available for proprietary reasons and have provided a contact point for accessing this data.

**Response 3: Thank you for the suggestion. According to your suggestion, we have modified in the section of “Support information”. For details, see the red font on page 26, lines 880-885.**

**Point 4:** Please include a separate caption for each figure in your manuscript.

**Response 4: Thanks for your suggestion. We have added a separate file for figure captions in our revised manuscript.**

**Response to Reviewer 1 Comments**

**Reviewer#1:**

**Point 1.** The concept of this study is interesting, but presentation of results is unclear. It would be better to explain the neural origin of each component (P1, P2, N330, especially LPP) and the significance of activity from each region (PO3, O1, POz, O2, PO4 for P1; P3 to P6 for P2; Fz and F1 for N330; CPz, CP1, and CP2 for LPP, also why the regions were selected) in the introduction, and then state what the authors wanted to clarify in this study.

**Response 1: Thank you for your great suggestion. According to your suggestion, we have modified in the section of “Introduction”. For details, see the red font on page 3-4, lines 89-110.**

**Point 2.** Grand average works well for checking the overall waveform, but is not suitable for statistical comparisons of each component. The amplitude and latency of each component of the ERP should be described by a box-and-whisker or bar graph. Since there appears to be more difference in amplitude than in latency for P1, the mean and standard deviation should be plotted to clarify the difference.

**Response 2: Thanks for your suggestion. We totally agree that statistical plots are also needed to illustrate the differences found in the tests. We added several boxplots for the reaction time result, as well as the latency results for the P1, P2, and N330. Please refer to the new plots.**

**Point 3.** In the results, the authors should explain why there is a difference between P3 and P5 sites in the peak amplitude of P2 component, and should also state what the difference means. Similarly, describe the behavioral data using figures.

**Response 3: Thank you for your good suggestion. According to your suggestion, we have modified in the section of “Discussion”. For details, see the red font on page 22, lines 759-761. We also add a boxplot fir the reaction time results. Please refer to the new Fig.2**

**Point 4.** The comparison of motion characteristic especially between “Global” and “Local” may have been influenced more by confusability than by the amount of information; if the Local figure were also placed so that the figure was centered rather than up and down, it would not be equivalent, but the confusability could be closer to that of the whole body. This is just a comment and please consider that next time. P1 should discuss which site caused the difference in latency and what its significance is. Since P1 Peak is expressed at each site, we should discuss what this component indicates.

**Response 4: Thank you for your good suggestion. According to your suggestion, we have modified in the section of “Discussion”. For details, see the red font on page 21, lines 739-738.**

**Minor comments**

**Point 1.** L239-240 It says that there is a significant difference in post-hoc P1 peak latency, but does not indicate which site.

**Response: Thanks for your suggestion. According to your suggestion, we have modified in the section of “Electrophysiological Data”. For details, see the red font on page 12, lines 365-366.**

**Point 2.** Figure Legend does not indicate which the region is indicated.

**Response 2: Thanks for your suggestion. We re-arranged the electrode label (legend) in the plots and marked the electrodes on the topographic map and the captions.**

**Response to Reviewer 2 Comments**

**Reviewer #2:**

**Point 1.** Line. 42, Rephrase the sentence. “In the current study, we examined the effects of acute psychological stress on the perceptual processing of biological motion and attention as a mechanism of these effects”

**Response 1: Thank you for your good suggestion. According to your suggestion, we have modified on the page 2, line 55.**

**Point 2.** Line 45, Two sentences redundant: “Acute stress can be induced by the improved Montreal Imaging Stress Task (MIST) [7]. The improved MIST task uses multiplication to induce acute stress.”

**Response 2: Thank you for your good suggestion. According to your suggestion, we have modified on the page 2, line 59.**

**Point 3.** Line 67 Please rephrase “In this study, the dynamic sequence diagram of biological light spots was used to study biological motion perception ability”

**Response 3: Thank you for your good suggestion. According to your suggestion, we have modified on the page 3, line 86.**

**Point** 4. The volunteer college students, did they receive credit, or money? “Inversion effect” is not clearly described.

**Response 4: Thank you for your good suggestion. According to your suggestion, we have modified on the page 5, lines 156-158 and page 1, lines 17-18.**

**Point 5.** Line 150 please rephrase “The mental arithmetic items in the formal experiment (10 trials) were different from those in the formal experiment”.

**Response 5: Thank you for your good suggestion. According to your suggestion, we have modified on the page 7, line 209.**

**Point 6.** Line 166, please modify the sentence “In the formal experiment, the red arrow will not be marked.”

**Response 6: Thank you for your good suggestion. According to your suggestion, we have modified on the page 8, lines 236-237.**

**Point 7.** Line 186, please rephrase “The length and bad interval free for ICA interval were both 50 s.”

**Response 7: Thank you for your good suggestion. According to your suggestion, we have modified on the page 9, line 303.**

**Point** 8. Figure legends are not descriptive enough, they should be more comprehensive. This makes the figures hard to decipher.

**Response 8: Thank you for your good suggestion. According to your suggestion, we have modified from Fig.1 to Fig.8. The descriptive content was added in the figure legends.**

**Point 9.** Line 465, There needs a better explanation than the current for LPP: “We can conclude that inhibition persistence was longer in the global upright and local inverted walking conditions than in the local upright and global inverted walking conditions.”

**Response 9: Thank you for your good suggestion. According to your suggestion, we have modified on the pages 23-24, lines 803-819.**
